# Empagliflozin Preserves Cardiomyocyte Structural Homeostasis via the Stabilization of the Integrin α5–Desmocollin-2 Adhesion Axis in Sepsis-Induced Cardiomyopathy

**DOI:** 10.3390/cells14181452

**Published:** 2025-09-16

**Authors:** Gan Qiao, Yongxiang Lu, Jianping Wu, Chunyang Ren, Minghua Liu, Sicheng Liang, Chunxiang Zhang

**Affiliations:** 1 Department of Pharmacology, School of Pharmacy, Southwest Medical University, Luzhou 646000, China; dqz377977905@swmu.edu.cn (G.Q.); luyx0807@163.com (Y.L.); 17865681795@163.com (J.W.); swmurcy@163.com (C.R.); minghualiu@swmu.edu.cn (M.L.); 2 Central Nervous System Drug Key Laboratory of Sichuan Province, Southwest Medical University, Luzhou 646000, China; 3 Department of Gastroenterology, The Affiliated Hospital of Southwest Medical University, Luzhou 646000, China; liangpharm@swmu.edu.cn; 4 Department of Cardiology, The Affiliated Hospital of Southwest Medical University, Institute of Cardiovascular Research, Key Laboratory of Medical Electrophysiology, Ministry of Education, Basic Medicine Research Innovation Center for Cardiometabolic Diseases, Nucleic Acid Medicine of Luzhou Key Laboratory, Southwest Medical University, Luzhou, Sichuan 646000, China

**Keywords:** empagliflozin, sepsis, cardiomyopathy, ITGA5, DSC2

## Abstract

Sepsis-induced cardiomyopathy is a life-threatening complication lacking targeted therapies. While empagliflozin (Empa), a sodium-glucose cotransporter 2 (SGLT2) inhibitor, confers robust cardioprotection, its specific efficacy in treating sepsis-induced cardiomyopathy and the Empa mechanisms remain poorly defined, limiting its targeted therapeutic use. In this study, we investigated Empa’s effects and its novel mechanisms in a murine lipopolysaccharide (LPS)-induced model of septic cardiomyopathy. Empa pre-treatment effectively prevented LPS-induced cardiac dysfunction, preserving ejection fraction and mitigating myocardial injury (assessed by histology and ELISA) and fibrosis. Transcriptomic analysis revealed that Empa’s protective effects were profoundly linked to the preservation of cardiomyocyte cytoskeletal pathways, alongside its anti-inflammatory actions. The results indicate that LPS induced a pathological dissociation of the matrix protein Integrin α5 (ITGA5) from the cell–cell adhesion protein Desmocollin-2 (DSC2), a structural disruption completely abrogated by Empa in vivo. This ITGA5-DSC2 stabilization was further confirmed to be a cardiomyocyte-intrinsic effect, recapitulated in vitro in both neonatal mouse cardiomyocytes and human AC16 cells. Building on this mechanistic insight, a computational design was successfully employed to develop 13 novel helical protein binders specifically targeting the ITGA5, yielding candidates with favorable structural properties as potential therapeutic leads. These findings establish the cardiomyocyte structural homeostasis via the ITGA5-DSC2 adhesion axis as a novel, key SGLT2-independent mechanism for empagliflozin’s cardioprotection, revealing promising new therapeutic approaches for sepsis-induced cardiomyopathy.

## 1. Introduction

Sepsis-induced cardiomyopathy is a devastating complication of systemic inflammation that significantly increases mortality in septic patients [1]. While heart failure is a major global health burden, existing therapeutic strategies often provide limited benefit in the acute and complex setting of sepsis, failing to address the specific molecular injuries that drive cardiac dysfunction [2]. This therapeutic gap underscores an urgent need for novel cardioprotective agents that target the fundamental mechanisms preserving cardiomyocyte integrity during severe inflammatory stress [3], offering new hope for a condition with persistently high lethality.

The sodium-glucose cotransporter 2 (SGLT2) inhibitor empagliflozin (Empa) has emerged as a powerful cardioprotective agent, with benefits extending far beyond its original role in glycemic control for type 2 diabetes mellitus (T2DM). Mounting evidence from landmark clinical trials such as EMPA-REG OUTCOME, DAPA-HF, and EMPEROR-Reduced demonstrates significant reductions in cardiovascular mortality, hospitalizations for heart failure, and progression of chronic kidney disease in patients with and without diabetes, suggesting these effects are largely independent of its glucose-lowering activity [4,5,6,7]. While various SGLT2-independent mechanisms have been proposed, including hemodynamic effects (natriuresis, osmotic diuresis, reduced preload/afterload), metabolic shifts (increased ketone body utilization), sympatholysis, immunomodulation, and improved hematocrit, the comprehensive molecular underpinnings of gliflozin’s profound cardioprotective profile remain incompletely characterized [6,8,9,10]. Concurrently, research has highlighted the critical importance of myocardial architecture, with cell adhesion proteins being indispensable for maintaining cardiac structural and functional integrity. Specifically, Integrin α5 (ITGA5), a key mediator of cell–matrix adhesion, and Desmocollin-2 (DSC2), an essential component of desmosomal cell–cell junctions, play crucial roles in maintaining cardiomyocyte cohesion [11,12]. Pathogenic mutations in DSC2, for instance, are a known cause of arrhythmogenic cardiomyopathy (ARVC), a severe inherited heart muscle disease characterized by progressive myocyte loss and fibrofatty replacement, leading to significant risk of sudden cardiac death [11]. Similarly, the dysregulation of ITGA5 has not only been implicated in the progression of cardiac fibrosis and adverse ventricular remodeling but has also emerged as a novel biomarker in the pathogenesis of other devastating cardiovascular conditions such as acute aortic dissection [12]. The disruption of these critical adhesion complexes is a central feature of multiple cardiovascular diseases, suggesting that their preservation represents a therapeutic strategy for protecting the heart.

In this study, we want to find the mechanism of the cardioprotective effects of Empa during sepsis. Using a lipopolysaccharide (LPS)-induced model of septic cardiomyopathy in mice, we demonstrate that Empa pre-treatment effectively prevents cardiac dysfunction, myocardial injury, and fibrosis. Mechanistically, we reveal that LPS causes a pathological dissociation of ITGA5 from DSC2 in cardiomyocytes, a structural disruption that Empa completely prevents, a finding confirmed to be intrinsic to cardiomyocytes both in vivo and in vitro. Furthermore, transcriptomic analysis indicated that Empa’s protective effects were linked to the preservation of cardiomyocyte cytoskeletal pathways, reinforcing the importance of structural integrity. Building on this discovery, we employed computational methods to design novel helical protein binders for the ITGA5, presenting a promising new therapeutic strategy. Our findings thus identify the ITGA5-DSC2 axis as a key mechanism behind Empagliflozin’s cardioprotective action in sepsis, offering a novel explanation for this drug’s remarkable properties and opening a new avenue for treating this condition.

## 2. Materials and Methods

### 2.1. Reagents

Empagliflozin (Cat. No. 864070-44-0, TargetMol, Wellesley Hills, MA, USA) was dissolved in sterile saline. Lipopolysaccharide (LPS) from Escherichia coli O111:B4 (Cat. No. HY-D1056, MedChemExpress, Monmouth Junction, NJ, USA) [13] was dissolved in sterile saline.

### 2.2. Animal Model and Experimental Design

All animal experiments were approved by the Institutional Animal Care and Use Committee of Southwest Medical University (Protocol No. 20221117-054). Male wild-type C57BL/6 mice (8 weeks old, GemPharmatech, Nanjing, China) were housed under a 12 h light/dark cycle with ad libitum access to food and water, before being randomly assigned to Control (n = 8), LPS (n = 12), or LPS+EMPA (n = 12) groups. Beginning on Day 8, the LPS+EMPA group received empagliflozin (30 mg/kg by oral gavage) once daily for 7 consecutive days [14]. On Day 15 (after the pretreatment period), the LPS and LPS+EMPA groups were received with a single intraperitoneal injection of LPS (6 mg/kg) [15], while Control mice received an equivalent volume of normal saline. On Day 16 (24 h post-LPS), cardiac function was assessed by transthoracic echocardiography as previously described [16], after which mice were euthanized and tissues collected [17]. This design uses a single-dose, acute endotoxemia model that produces a transient septic insult; while it does not replicate the protracted, complex course of human sepsis, it is widely used to dissect early inflammatory and structural mechanisms of sepsis-induced cardiomyopathy and to evaluate preventive interventions.

### 2.3. Echocardiography

Transthoracic echocardiography was performed on mice anesthetized with 1% isoflurane using a high-resolution small animal ultrasound imaging system (Vevo 3100, FUJIFILM VisualSonics, Inc., Toronto, ON, Canada) equipped with an MX400 linear array transducer (20–46 MHz). B-mode long-axis images were acquired for anatomical overview. M-mode images were recorded from the parasternal short-axis view at the level of the papillary muscles. Representative images were obtained at the mid-ventricular level of the ventricular to ensure consistent anatomical sampling across groups. Left ventricular anterior wall (LVAW), posterior wall (LVPW), and internal diameter (LVID) were measured during both systole and diastole from the left ventricular M-mode recording. Left ventricular ejection fraction (EF) and fractional shortening (FS) were calculated from these parameters (LVAW, LVPW, and LVID) using the system’s automated algorithm. Diastolic function was assessed using pulsed-wave Doppler echocardiography from the apical four-chamber view, with the sample volume placed at the tip of the mitral valve leaflets to measure early (E) and late (A) diastolic filling velocities. The ratio of early to late filling velocity (E/A ratio) was calculated as an index of diastolic function.

### 2.4. ELISA

Blood samples were collected via cardiac puncture into EDTA-coated tubes. Serum was separated by centrifugation at 2500 rcf for 15 min at 4 °C. Serum levels of cardiac troponin T (cTnT) and C-reactive protein (CRP) were quantified using commercial enzyme-linked immunosorbent assay (ELISA) kits (cTnT: Cat. No. orb565454, Biorbyt, Wuhan, China; CRP: Cat. No. orb219654, Biorbyt, Wuhan, China) according to the manufacturer’s instructions.

### 2.5. Histology and Immunohistochemistry (IHC)

Hearts were excised, rinsed with PBS, fixed in 4% paraformaldehyde for 24 h, and embedded in paraffin. Sections (5 μm) were stained with Hematoxylin and Eosin (H&E) for general morphology and Masson’s trichrome for fibrosis assessment using a commercial kit (Cat. No. G1006, Servicebio, Wuhan, China). For IHC, sections were subjected to antigen retrieval in citrate buffer (pH 6.0). Sections were blocked with 5% goat serum and incubated overnight at 4 °C with the following primary antibodies: anti-α-smooth muscle actin (α-SMA) (Cat. No. HY-P80484, MedChemExpress, 1:200), anti-F4/80 (Cat. No. HY-P80665, MedChemExpress, 1:200), anti-Integrin α5 (ITGA5) (Cat. No. HY-P80416, MedChemExpress, 1:200), and anti-Desmocollin-2 (DSC2) (Cat. No. 13876-1-AP, Proteintech Group, Inc., Rosemont, IL, USA, 1:200). After incubation with appropriate HRP-conjugated secondary antibodies (Cat. No. GB23301, Servicebio, 1:200), signals were visualized using a DAB substrate kit (Cat. No. G1212, Servicebio). Images were captured with a light microscope (E100, Nicon) and quantitative analysis was performed using ImageJ software (Version 1.48q).

### 2.6. Bioinformatic Analysis of Transcriptomic Datasets

To corroborate the primary findings, a comparative bioinformatic analysis of a relevant public dataset retrieved from the Gene Expression Omnibus (GEO) was performed: GSE267388 (mRNA expression profiles in hearts from a sepsis-induced myocardial dysfunction mouse model). Raw count data and associated metadata were processed using a standardized pipeline. Differential gene expression analysis between LPS-treated and control groups was conducted using the DESeq2 package (v1.40.2). Gene identifiers were annotated using the database. Genes were considered significantly dysregulated if they met the following statistical criteria: base mean > 10, absolute log2 fold change > 1, and a *p*-value < 0.05.

To elucidate the biological functions of the differentially expressed genes, pathway and gene-concept network analysis was performed using the clusterProfiler package (v4.8.3). This approach allowed for the identification and visualization of key structural pathways, particularly the “Cytoskeleton in muscle cells” pathway, that are disrupted by LPS challenge. The consistent identification of cytoskeletal dysregulation as a central pathogenic hub across independent datasets provided the validation of the primary mechanistic hypothesis.

### 2.7. RNA Sequencing and Bioinformatics Analysis

Total RNA was extracted from heart tissues (approximately 30 mg) using TRIzol^®^ reagent (R480101, Magen Biotechnology Co., Ltd., Guangzhou, China) following the manufacturer’s instructions. RNA quality was assessed using a Nanodrop ND-2000 system (A260/A280 ratio) and integrity was confirmed with an Agilent Bioanalyzer 4150 system using the RNA Nano 6000 Assay Kit, with only samples having RNA integrity numbers (RIN) > 8.0 selected for sequencing. Paired-end mRNA sequencing libraries were prepared using the ABclonal mRNA-seq Lib Prep Kit following standard protocol: mRNA purification from 1 μg total RNA using oligo (dT) magnetic beads, fragmentation, first-strand cDNA synthesis with random hexamer primers and reverse transcriptase, second-strand synthesis, end repair, A-tailing, and adapter ligation. Libraries were PCR-amplified, purified using the AMPure XP system (Beckman Coulter, Brea, CA, USA), quality-controlled on an Agilent Bioanalyzer 4150, and quantified via Qubit 4.0 Fluorometer (Life Technologies, CA, USA). After pooling in equimolar ratios, sequencing was performed on an Illumina NovaSeq 6000 platform with 2 × 150 bp paired-end configurations, generating an average of 30 million reads per sample.

The bioinformatic workflow began with the quality control of raw reads (filtering out reads with >60% bases having quality scores ≤ 25 and >5% undetermined bases), followed by alignment to the mouse reference genome (mm10) using HISAT2 (v2.2.1). Gene expression quantification was performed using FeatureCounts (v2.0.3) with FPKM values calculated based on gene length and read counts. Differential expression analysis was conducted using DESeq2 (v1.34.0) with significance thresholds of |log2FoldChange| > 1 and adjusted *p*-value < 0.05. Functional annotation included Gene Ontology enrichment (topGO v2.46.0, GO.db v3.14.0) and KEGG pathway analysis (KOBAS v3.0.3), with gene identifiers mapped using clusterProfiler (v4.8.3) and org.Mm.eg.db (v3.21.0). Results were visualized using Pearson correlation heatmaps (ComplexHeatmap v2.16.0), 3D PCA plots (plotly v4.10.4), and expression clustering using the TCseq method implemented in the ClusterGVis package (v0.2.1). For visualization and functional interpretation and differentially expressed genes were partitioned into four expression clusters with comprehensive heatmaps and integrated enrichment bar plots generated using the visCluster function to reveal biological processes modulated by Empa.

### 2.8. Cell Culture

Neonatal mouse cardiomyocytes (NMCMs) were isolated from 1–3-day-old C57BL/6 mice as previously described [18]. The human cardiomyocyte cell line AC16 (Meisen CTCC, Jinhua, China, Cat. No. CTCC-003-0014) was cultured in DMEM supplemented with 10% fetal bovine serum and 1% penicillin-streptomycin. Cells were cultured at 37 °C in a 5% CO2 incubator and treated with LPS (0.5 μg/mL) with or without Empa (10 μM) pre-treatment for 24 h.

### 2.9. Western Blotting

Heart tissues and cultured cells were lysed in RIPA buffer (Cat. No. PC101, Epizyme Biotech) containing protease and phosphatase inhibitors (Cat. No. P1260, Solarbio, Beijing, China). Protein concentrations were determined using a BCA assay kit (Cat. No. P0009, Beyotime, Haimen, China). Equal amounts of protein (20 μg) were separated by SDS-PAGE on 10% polyacrylamide gels, transferred to PVDF membranes (Cat. No. ISEQ00010, MilliporeSigma, Burlington, MA, USA), and blocked with 5% non-fat milk in TBST. Membranes were incubated overnight at 4 °C with primary antibodies against ITGA5 (Cat. No. HY-P80416, MedChemExpress, 1:1000), DSC2 (Cat. No. 13876-1-AP, Proteintech Group, Inc., Rosemont, IL, USA, 1:1000), and GAPDH (Cat. No. HY-P80137, MedChemExpress, 1:2000). After incubation with HRP-conjugated secondary antibodies (Cat. No. HY-P8001, MedChemExpress, 1:5000), bands were visualized using an ECL detection system (SQ201, Epizyme Biotech, Cambridge, MA, USA).

### 2.10. Computational Design of ITGA5 Binders

To computationally design novel helical binders targeting human integrin alpha-5 (ITGA5), an automated multistage pipeline was executed, beginning with the identification of a key binding hotspot on the ITGA5 target structure (PDB: 7NWL, chain A) [19]. The pipeline initiated the design process by generating 75-residue helical backbones using a ColabDesign hallucination protocol, which was explicitly constrained to this predefined target interface [20]. For each generated backbone, ProteinMPNN was then employed to design and score multiple candidate amino acid sequences, with the top-scoring sequence selected for comprehensive structural evaluation. The structure of the designed binder-target complex was predicted using AlphaFold2-multimer and subsequently refined with the FastRelax protocol in PyRosetta to minimize steric clashes and optimize the binding geometry. To ensure the selection of only the highest-quality candidates, the resulting designs were subjected to a rigorous filtering cascade based on multiple biophysical and structural criteria. A design was required to demonstrate high prediction confidence (mean pLDDT > 85, interface pTM > 0.7), close structural fidelity to the intended model (Cα-RMSD < 4.0 Å), excellent shape complementarity at the interface (>0.6), and high conformational stability, confirmed by a low interface RMSD (<2.0 Å) and a minimal structural change between the bound and unbound binder states (self-consistency RMSD < 2.0 Å). Only designs that successfully passed all of these stringent filters were retained for final analysis. The geometric accuracy of the binding interface for each complex were quantitatively evaluated using the boltz-2 method [21]. For detailed structural analysis and the generation of high-quality molecular illustrations, the coordinates of the predicted complexes were rendered using UCSF ChimeraX [22].

### 2.11. Statistical Analysis

Data are presented as mean ± SEM. Statistical significance between groups was determined using one-way ANOVA followed by Tukey’s post hoc test using GraphPad Prism (Version 9.0, GraphPad Software, La Jolla, CA, USA). A *p*-value < 0.05 was considered statistically significant.

## 3. Results

### 3.1. Empagliflozin Ameliorates LPS-Induced Cardiac Dysfunction and Myocardial Injury

**Figure 1:**
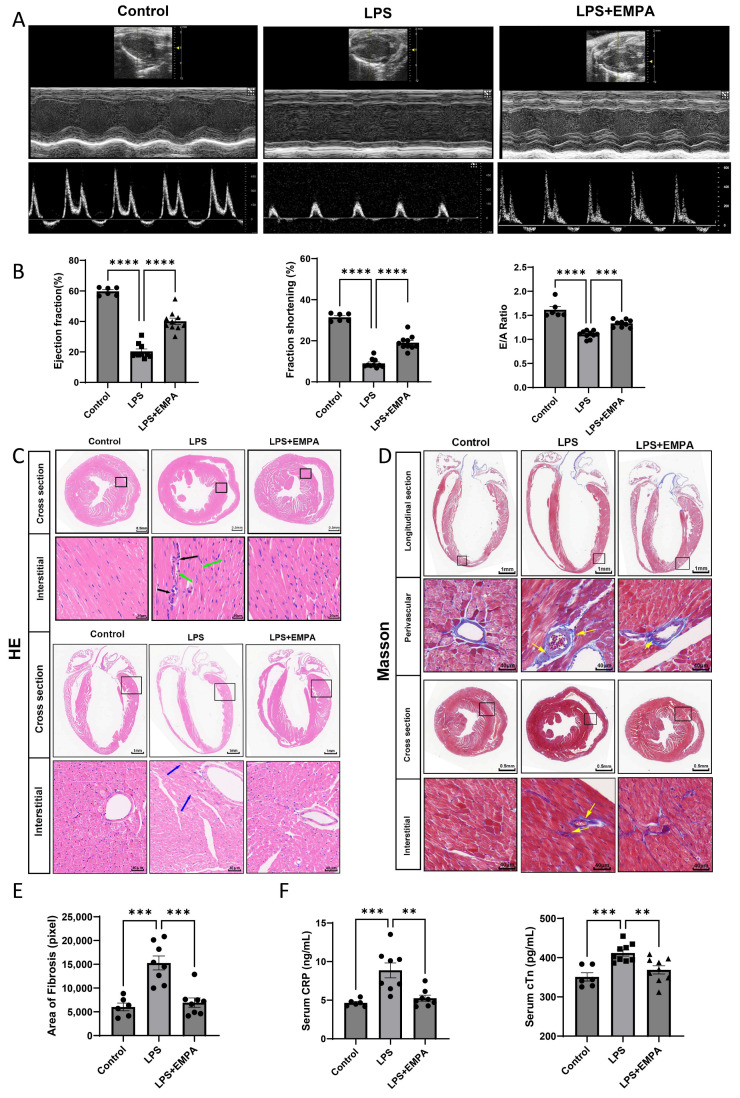
Empagliflozin ameliorates LPS-induced cardiac dysfunction and myocardial injury. (**A**) Echocardiography panels: top, B-mode long-axis image; middle, M-mode from the parasternal short-axis at the papillary muscle level; bottom, pulsed-wave Doppler of transmitral inflow (E and A waves). (**B**) Quantification of cardiac function parameters: Ejection Fraction (EF), Fractional Shortening (FS), and E/A Ratio. (**C**) Representative images of Hematoxylin and Eosin (H & E) staining of myocardial tissue sections showing inflammatory infiltration. Longitudinal and cross sections are shown with a scale bar of 1 mm. Magnified views of perivascular and interstitial regions are shown with a scale bar of 20 µm; green arrows indicate inflammatory cells. (**D**) Representative images of Masson’s trichrome staining revealing myocardial fibrosis. Longitudinal sections are shown with a scale bar of 1 mm, and cross sections with a scale bar of 0.5 mm. Magnified views of perivascular and interstitial regions are shown with a scale bar of 40 µm; yellow arrows indicate collagen deposition (fibrotic areas). (**E**) Quantification of the fibrotic area from Masson’s trichrome staining. (**F**) Serum concentrations of cardiac troponin T (cTnT) and C-reactive protein (CRP) measured by ELISA. Data are presented as mean ± SEM. Statistical analysis was performed using one-way ANOVA. **** *p* < 0.0001, *** *p* < 0.001, ** *p* < 0.01.

The cardioprotective potential of Empa in endotoxemia was investigated by assessing cardiac performance using echocardiography. As illustrated in Figure 1A, lipopolysaccharide (LPS) administration induced severe cardiac dysfunction. Quantitative analysis confirmed this, revealing a catastrophic decline in systolic function, with ejection fraction (EF) plummeting from a control mean of 59.69% ± 2.71% to 20.38% ± 2.35% (*p* < 0.0001) and fractional shortening (FS) decreasing from 31.41% ± 1.46% to 8.18% ± 1.22% (*p* < 0.0001). Diastolic function was similarly impaired, as evidenced by a significant reduction in the E/A ratio from 1.62 ± 0.05 to 1.10 ± 0.06 (*p* < 0.001). Pre-treatment with Empa markedly reversed these deficits, restoring EF to 40.08% ± 2.81% and FS to 19.05% ± 1.50% (both *p* < 0.0001 vs. LPS), and significantly improving the E/A ratio to 1.33 ± 0.05 (*p* < 0.001 vs. LPS) (Figure 1B). Detailed echocardiographic parameters are presented in Table S1. This functional recovery was directly correlated with a reduction in myocardial tissue damage. Histological examination by H&E staining showed that LPS induced significant inflammatory cell infiltration, effects that were visibly attenuated by Empa (Figure 1C). Furthermore, Masson’s trichrome staining revealed extensive interstitial fibrosis in the LPS group, which was quantified as a nearly threefold increase in fibrotic area compared to controls (*p* < 0.01); Empa pre-treatment significantly reduced this fibrotic response (*p* < 0.05 vs. LPS) (Figure 1D,E). These structural findings were substantiated by serological markers of cardiac injury and inflammation. ELISA results demonstrated that the LPS-induced surge in serum cardiac troponin T (cTnT) was significantly blunted by Empa (*p* < 0.01), as was the elevation in C-reactive protein (CRP) (*p* < 0.05) (Figure 1F). Collectively, these data provide convergent evidence from functional, structural, and molecular assays that Empa ameliorates LPS-induced cardiac dysfunction by preserving myocardial integrity and attenuating the inflammatory and fibrotic response.

### 3.2. Empagliflozin Attenuates the Pro-Inflammatory and Pro-Fibrotic Cellular Response in the Myocardium

**Figure 2:**
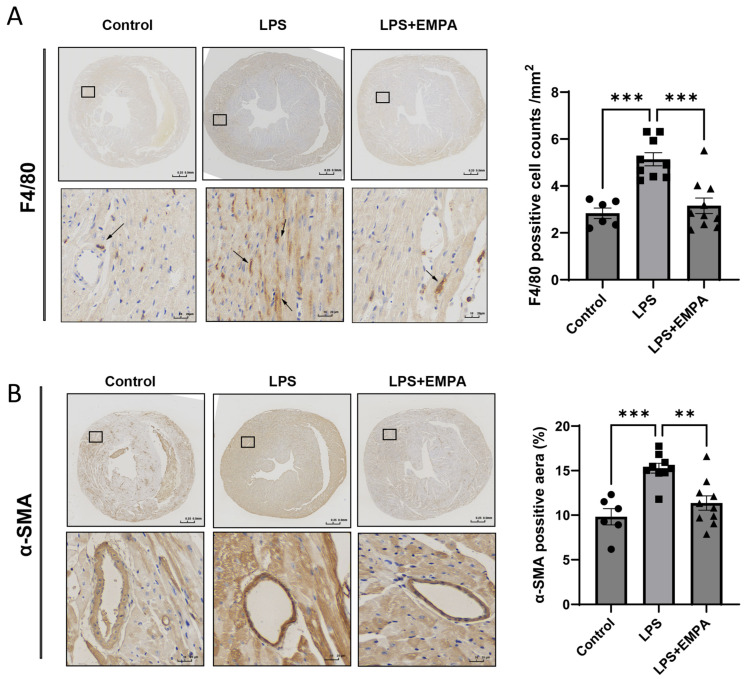
Empagliflozin attenuates the pro-inflammatory and pro-fibrotic cellular response in the myocardium. (**A**) Representative immunohistochemical staining and quantification of the macrophage marker F4/80 in myocardial tissue sections. Scale bars for whole heart sections are 0.25 mm, and for magnified views are 20 µm. Black arrows indicate F4/80 positive macrophages. (**B**) Representative immunohistochemical staining and quantification of the myofibroblast marker α-smooth muscle actin (α-SMA) in myocardial tissue sections. Scale bars for whole heart sections are 0.25 mm, and for magnified views are 20 µm. Data are presented as mean ± SEM. Statistical analysis was performed using one-way ANOVA. *** *p* < 0.001, ** *p* < 0.01.

Given that the functional protection conferred by Empa was associated with a marked reduction in tissue fibrosis, the organ-level outcome was driven by the modulation of underlying inflammatory and fibrotic cellular cascades. To test this, inflammatory cell infiltration was quantified using immunohistochemistry for the macrophage marker F4/80. In response to the LPS challenge, the density of F4/80-positive macrophages in the myocardial interstitium increased dramatically, from a baseline of 2.85 ± 0.46 cells/mm^2^ to 5.41 ± 0.45 cells/mm^2^ (*p* < 0.001). Empa pre-treatment potently suppressed this inflammatory influx by nearly 50%, reducing macrophage density to 3.51 ± 0.4 cells/mm^2^ (*p* < 0.001 vs. LPS; Figure 2A). Since macrophage-led inflammation is a key driver of fibroblast-to-myofibroblast transition, the activation of these pro-fibrotic cells was subsequently assessed by staining for α-smooth muscle actin (α-SMA). The α-SMA-positive tissue area, indicative of myofibroblast abundance, increased in the LPS group (from 9.82% ± 1.45% to 15.28% ± 1.12%, *p* < 0.001). Empa administration abrogated this response, reducing the α-SMA-positive area to 11.36% ± 0.99% (*p* < 0.01 vs. LPS; Figure 2B). The strong correlation between the suppression of macrophage infiltration and the reduction in myofibroblast activation provides evidence that Empa’s anti-fibrotic benefit is mechanistically linked to its potent anti-inflammatory action at the cellular level.

### 3.3. Unbiased Transcriptomic Profiling Reveals Cytoskeletal Integrity as a Core Target of Empagliflozin

**Figure 3:**
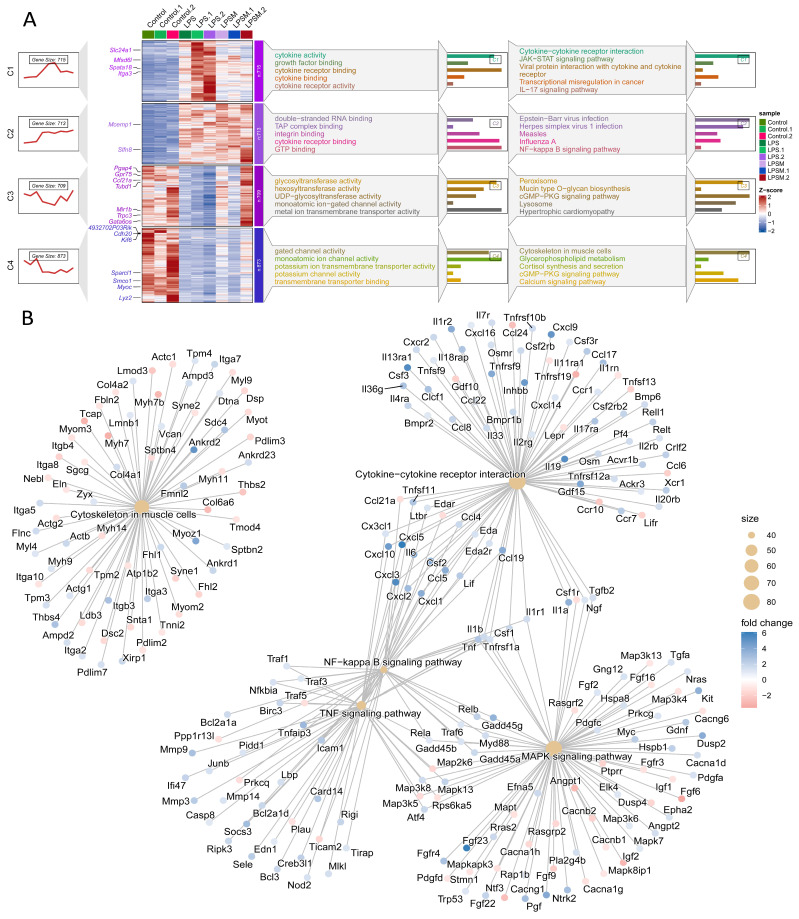
Unbiased transcriptomic profiling identifies cytoskeletal integrity as a core target of empagliflozin. (**A**) Multi-panel analysis of differentially expressed genes from cardiac tissue of Control, LPS, and LPS+Empa groups, partitioned into four clusters. Analysis includes expression trends, gene expression heatmap, and Gene Ontology (GO) and KEGG pathway enrichment for each cluster. (**B**) Gene-concept network (cnet) analysis of enriched KEGG pathways, visualizing the functional relationships between gene clusters. The “Cytoskeleton in muscle cells” pathway is highlighted as a distinct node from inflammatory pathways like TNF signaling and cytokine–cytokine receptor interaction.

To uncover a primary mechanism for Empa cardioprotection, we conducted transcriptomic profiling, which revealed that preserving cytoskeletal integrity is a core therapeutic target, distinct from its known anti-inflammatory effects. Unbiased whole-transcriptome RNA sequencing of cardiac tissue from control, LPS, and LPS+Empa groups identified 3010 expressed genes, 2424 (80.5%) of which were normalized by Empa. As illustrated in the comprehensive multi-panel analysis in Figure 3A, these differentially expressed genes were partitioned into four clusters to systematically characterize their biological significance. The visualization presents four analytical lanes: the first showing the expression trend for each cluster, the second displaying a detailed gene expression heatmap across all samples, the third presenting Gene Ontology (GO) enrichment, and the fourth depicting KEGG pathway enrichment. While this analysis confirmed Empa’s potent suppression of canonical inflammatory and cytokine signaling pathways, such as the NF-kappa B and JAK-STAT pathways evident in the enrichment results, it critically unveiled a previously unrecognized, dominant non-inflammatory gene signature as a primary target of the drug. The most significant of these was the KEGG pathway “Cytoskeleton in muscle cells”. The integrity of the cardiomyocyte cytoskeleton is an indispensable determinant of cardiac function, providing the structural scaffold for cellular shape and mechanical force transmission, and its disruption is a fundamental mechanism underlying myocardial dysfunction. The significant enrichment of this pathway demonstrates that Empa’s established SGLT2-independent cardio protection extends beyond systemic or anti-inflammatory effects to directly preserve this vital cellular infrastructure. This conclusion was further solidified by the KEGG pathway concept network (cnet) analysis in Figure 3B, which strikingly visualized the “Cytoskeleton in muscle cells” gene cluster as a large, functionally distinct node, clearly separate from the interconnected inflammatory pathways like TNF signaling and cytokine–cytokine receptor interaction. This pivotal discovery provides compelling evidence that Empa’s therapeutic effect is rooted in a dual mechanism involving not only immunomodulation but also the direct preservation of the cardiomyocyte’s fundamental structural framework.

### 3.4. Bioinformatic Validation Pinpoints the Cytoskeleton in the Muscle Cells Pathway as a Central Pathogenic Hub

**Figure 4:**
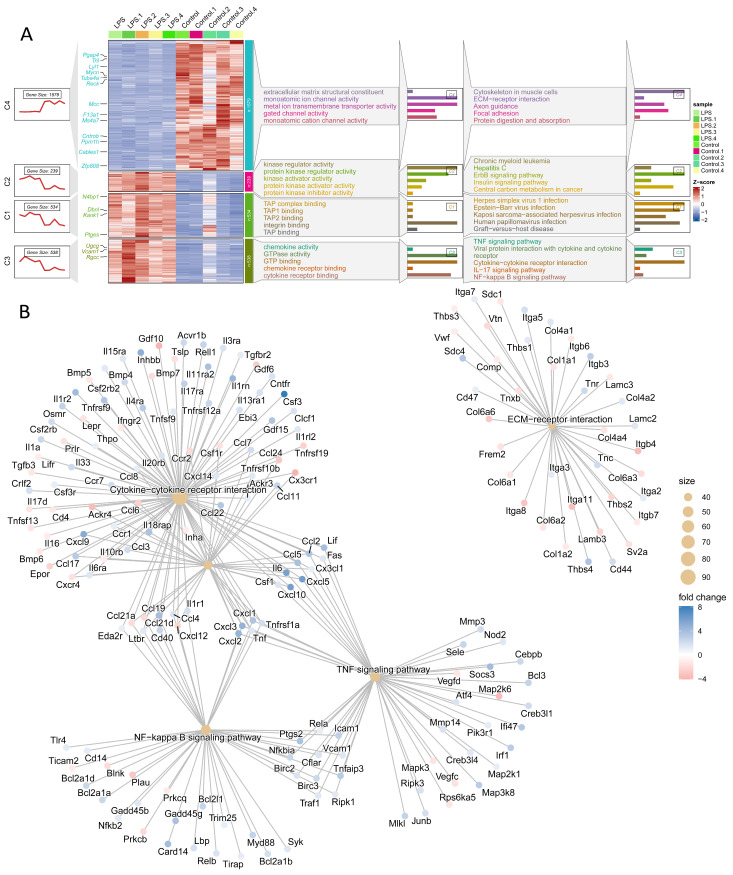
Bioinformatic validation on an independent dataset pinpoints the “Cytoskeleton in muscle cells” pathway as a central pathogenic hub. (**A**) Re-analysis of a publicly available transcriptomic dataset (GSE267388) from LPS-challenged hearts using a multi-panel format showing cluster expression trends, gene expression heatmap, and GO and KEGG pathway enrichment. (**B**) Gene-concept network visualizing the hierarchical organization of dysregulated pathways, highlighting the cytoskeletal/ECM-receptor module as distinct from the inflammatory cascade.

To validate the primary findings and confirm that structural dysregulation is a central pathogenic event, a bioinformatic analysis was performed on an independent, publicly available dataset of LPS-challenged hearts (GSE267388), which successfully pinpointed the “Cytoskeleton in muscle cells” pathway as a central hub. Using an identical computational workflow, this external dataset was visualized in a comprehensive multi-panel format (Figure 4A) presenting four analytical lanes: the first showing cluster expression trends, the second a gene expression heatmap, the third displaying Gene Ontology (GO) enrichment, and the fourth depicting KEGG pathway enrichment. This re-analysis provided powerful corroboration, confirming that beyond the expected inflammatory signature (TNF signaling, NF-κB signaling), LPS profoundly disrupts gene networks essential to cardiac architecture. Crucially, the KEGG enrichment analysis identified the “Cytoskeleton in muscle cells” pathway as the most significantly enriched non-inflammatory hub dysregulated by LPS, alongside other key structural pathways including “ECM-receptor interaction,” “Focal adhesion,” and those associated with hypertrophic and dilated cardiomyopathy. This finding was further substantiated by the gene-concept interaction network in Figure 4B, which elucidated the hierarchical organization of these pathways. The network map visualized the cytoskeletal ECM-receptor components as a prominent and distinct structural module, clearly separate from the interconnected inflammatory cascade. Of particular significance, the “ECM-receptor interaction” pathway exhibited extensive connectivity with multiple integrin-coding genes, including Itga5, reinforcing the importance of cell–matrix adhesion. The identification of this conserved cytoskeletal signature across independent transcriptomic datasets provides compelling evidence for the central role of structural protein dysregulation in sepsis-induced cardiomyopathy, thereby validating our focus on specific molecular constituents of the cardiomyocyte cytoskeleton.

### 3.5. LPS Induces a Pathological Inversion of the Physiological ITGA5-DSC2 Adhesion Axis

**Figure 5:**
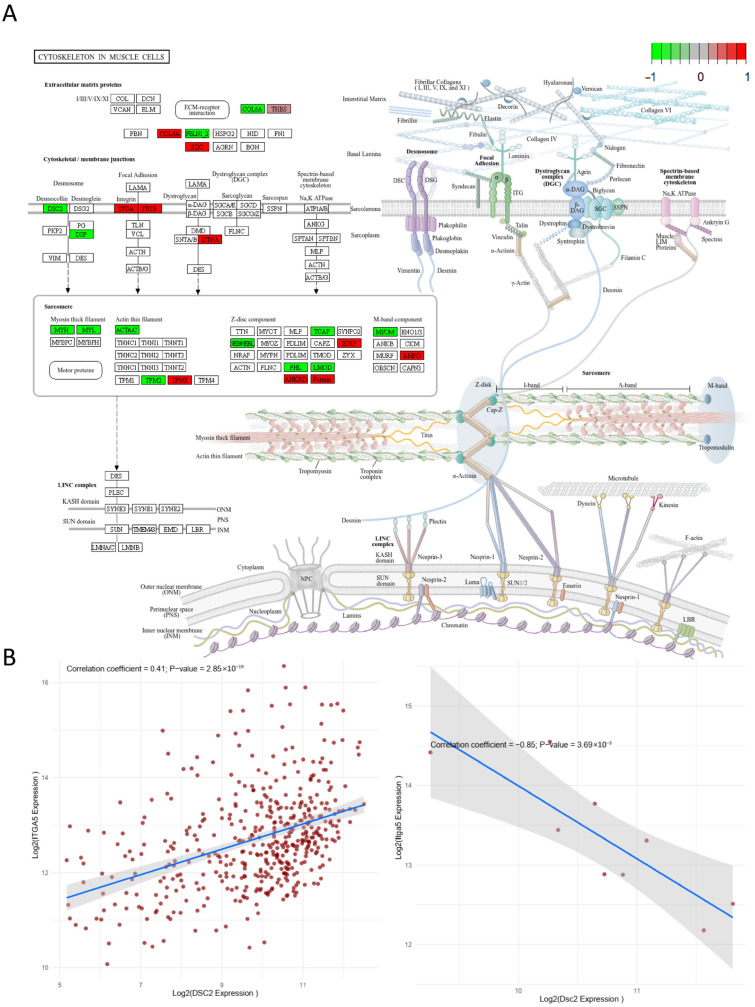
LPS induces a pathological inversion of the physiological ITGA5-DSC2 adhesion axis. (**A**) Pathview visualization of the KEGG “Cytoskeleton in muscle cells” pathway, illustrating LPS-induced dysregulation of genes encoding structural proteins. (**B**) Correlation analysis of ITGA5 and DSC2 expression. **Left**: A significant positive correlation is observed in normal human heart tissue from the GTEx database (r = 0.41, *p* < 2.85 × 10^−19^). **Right**: A strong negative correlation is induced by LPS challenge in the experimental mouse model (r = −0.85, *p* < 3.69 × 10^−3^), demonstrating a pathological inversion of the relationship.

Guided by the convergent evidence from the transcriptomic and bioinformatic analyses, a detailed Pathview analysis of the “Cytoskeleton in muscle cells” KEGG pathway was performed (Figure 5A). This comprehensive pathway mapping revealed that LPS induced significant perturbations in multiple structural components essential for cardiomyocyte integrity, with the most pronounced dysregulation occurring at the interface between matrix and cell–cell adhesion complexes. Among the affected components, two molecules emerged as critical nodes in this pathological response: Integrin α5 (ITGA5), a cornerstone of matrix adhesion that links the extracellular matrix to the cytoskeleton, and Desmocollin-2 (DSC2), a vital component of intercellular desmosomes that mediates mechanical coupling between adjacent cardiomyocytes.

The coordinated expression and functional interplay of these two molecules represent a previously unrecognized homeostatic mechanism essential for cardiac structural integrity. Analysis of normal human heart tissue from the GTEx database revealed a significant positive correlation (r = 0.41, *p* < 2.85 × 10^−19^) between ITGA5 and DSC2 expression levels (Figure 5B, left), confirming their physiological co-regulation in healthy myocardium. In stark contrast, the LPS challenge in the experimental model pathologically inverted this relationship, inducing a strong negative correlation (r = −0.85, *p* < 3.69 × 10^−3^; Figure 5B, right) between these two adhesion molecules. This dramatic switch from a synergistic to an antagonistic relationship signifies a catastrophic uncoupling of the cell–matrix and cell–cell adhesion machinery, with ITGA5 being pathologically upregulated while DSC2 is simultaneously downregulated. The mathematical quantification of this inverted relationship provides evidence that the disruption of the ITGA5-DSC2 axis is not merely associated with septic cardiomyopathy but likely represents a core driving mechanism of cardiomyocyte structural and functional failure during sepsis.

### 3.6. Empagliflozin Prevents the Pathological Dysregulation of the ITGA5-DSC2 Axis at the Protein Level

**Figure 6:**
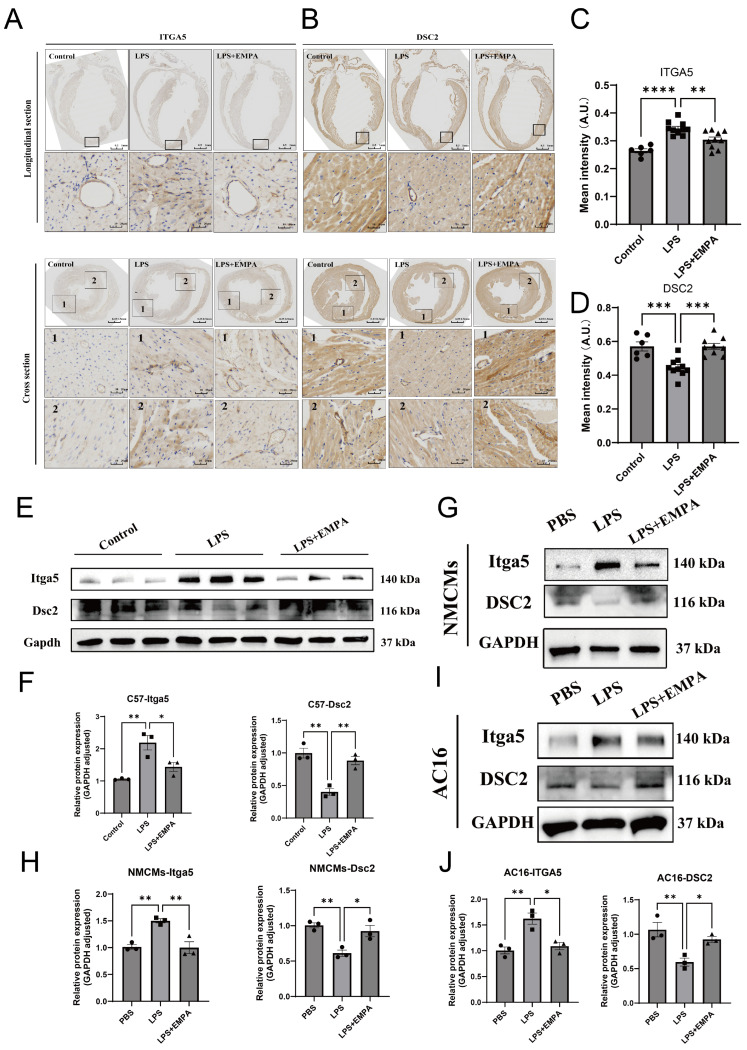
Empagliflozin prevents the pathological dysregulation of the ITGA5-DSC2 axis at the protein level. Representative immunohistochemical staining for ITGA5 (**A**) and DSC2 (**B**) in longitudinal and cross-sections of cardiac tissue from Control, LPS, and LPS+Empa groups. And the numbered boxes (1 and 2) indicate the regions from which the higher-magnification images were taken. Scale bars: whole-heart sections, 1 mm; higher-magnification views, 20 μm. Densitometric quantification of mean intensity for ITGA5 (**C**) and DSC2 (**D**) staining. Western blot analysis (**E**) and quantification (**F**) of Itga5 and Dsc2 protein levels in whole heart lysates from C57 mice. Western blot analysis (**G**) and quantification (**H**) of Itga5 and Dsc2 protein levels in primary neonatal mouse cardiomyocytes (NMCMs). Western blot analysis (**I**) and quantification (**J**) of ITGA5 and DSC2 protein levels in the human AC16 cardiomyocyte cell line. Data are presented as mean ± SEM. Statistical analysis was performed using one-way ANOVA. **** *p* < 0.0001, *** *p* < 0.001, ** *p* < 0.01, * *p* < 0.05.

A multi-tiered investigation provided conclusive evidence that Empa prevents the pathological dysregulation of the ITGA5-DSC2 axis at the protein level, thereby preserving fundamental cardiac structural integrity. The immunohistochemical analyses on cardiac tissue sections offered stark visual and quantitative proof of Empa’s protective effect was performance. As depicted in representative longitudinal and cross-sections (Figure 6A,B), an inflammatory challenge with LPS induced a pronounced, diffuse upregulation of ITGA5 protein throughout the myocardium, a stark contrast to its minimal expression in control tissue. Simultaneously, LPS triggered a marked reduction and delocalization of Desmocollin-2 (DSC2), a critical protein normally localized robustly at the intercalated disks to maintain vital cell–cell adhesion. This pathological shift was quantified through densitometric analysis, which confirmed the reciprocal dysregulation: LPS significantly increased ITGA5 mean intensity from a baseline of 0.26 to 0.34 (*p* < 0.0001) while concurrently decreasing DSC2 mean intensity from 0.57 to 0.45 (*p* < 0.001) (Figure 6C,D). Crucially, Empa pre-treatment effectively prevented this pathological structural remodeling. It significantly attenuated the LPS-induced dysregulation in ITGA5 (*p* < 0.01 vs. LPS) and, importantly, fully preserved DSC2 expression (*p* < 0.001 vs. LPS), safeguarding the heart’s structural framework. To further quantify these protein alterations and, critically, to ascertain if Empa’s protective mechanism is an intrinsic property of cardiomyocytes, the investigation transitioned to protein expression analyses. These analyses first confirmed the in vivo findings in whole heart lysates from C57 mice, showing a significant LPS-induced upregulation of Itga5 and downregulation of Dsc2 that were both significantly reversed by Empa co-treatment (Figure 6E,F). The pivotal mechanistic question was then addressed using two distinct in vitro cardiomyocyte models. In both primary neonatal mouse cardiomyocytes (NMCMs) (Figure 6G,H) and the human AC16 cardiomyocyte cell line (Figure 6I,J), stimulation with LPS alone was sufficient to recapitulate the pathological protein signature, significantly increasing ITGA5 and decreasing DSC2 levels. In both of these isolated systems, devoid of confounding systemic influences, concurrent treatment with Empa directly and effectively abrogated this pathological response, normalizing the expression of both ITGA5 and DSC2. This cohesive, multi-level validation provides conclusive evidence that Empa prevent the pathological dysregulation of the ITGA5-DSC2 adhesion axis at the protein level.

### 3.7. Computational Design of ITGA5 Binders as Potential Therapeutic Agents

To address the pathological dysregulation of ITGA5 in septic cardiomyopathy, a computational design pipeline was employed to develop novel molecular binders targeting this pathway. This initiative yielded 13 unique helical binders, each precisely 75 amino acids in length, engineered to engage ITGA5 with a high predicted affinity and specificity (Supplementary Table S2). All designed molecules successfully passed stringent filtering protocols established to ensure high quality.

Quantitative assessment of the design metrics revealed high structural confidence and favorable interface characteristics across the binder cohort. The designs exhibited an average predicted Local Distance Difference Test (pLDDT) score of 87.1 ± 1.7, signifying a high degree of confidence in their computationally predicted tertiary structures. The binding interface was characterized by an average interface pTM score of 0.76 ± 0.02 and an average interface RMSD of 1.51 ± 0.18 Å, indicating accurate and reproducible predictions of the binding mode. Furthermore, the designed binders are predicted to form stable and energetically favorable interactions, with an average calculated binding free energy (ΔG) of −101.68 ± 12.48 kcal/mol. The interfaces demonstrate robust steric complementarity, with an average shape complementarity score of 0.67 ± 0.02 and minimal post-relaxation clashes.

**Figure 7:**
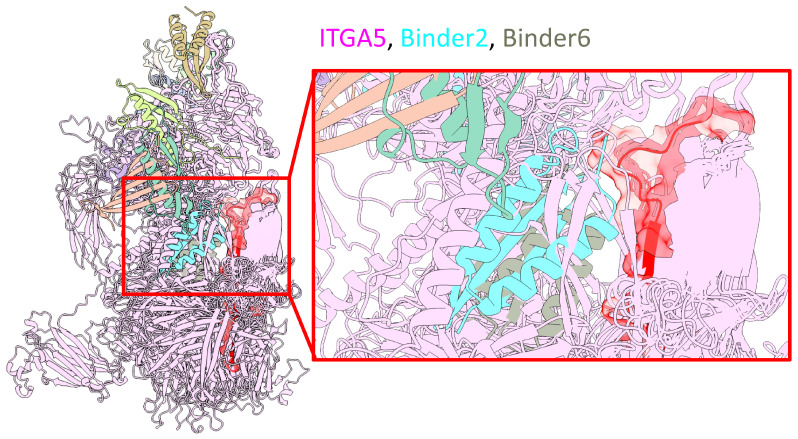
Computational design and structural modeling of de novo binders targeting ITGA5. Structural model of de novo designed helical binders in complex with ITGA5. Left: Overview showing the general interaction landscape of the binder cohort with the full ITGA5 protein (pink). Right: Magnified view of the binding interface, highlighting two exemplary binders, Binder-2 (cyan) and Binder-6 (red), docked into the predefined target hotspot region of ITGA5.

As shown in Figure 7, an overview model (left) shows the general interaction landscape of the designed binders with the full ITGA5 protein. A detailed, magnified view of the interface (right) highlights two exemplary candidates, Binder-2 (cyan) and Binder-6 (red), which are shown to successfully dock into the predefined target hotspot region on ITGA5 (pink). The accurate on-target engagement of these binders with the intended residues validates the computational design strategy. Based on this promising structural evidence, further investigation will be conducted to experimentally validate the functional effects of these binders. These de novo designed proteins represent a novel therapeutic modality that could potentially synergize with Empa by directly targeting ITGA5, offering a new strategy to preserve cardiac structural integrity during a septic challenge.

## 4. Discussion

This study delineates a novel, SGLT2-independent mechanism through which Empa confers direct cardioprotection against sepsis-induced cardiomyopathy, a condition that remains a major driver of mortality in critical care. Our findings establish that Empa pre-treatment robustly preserves cardiac function and mitigates myocardial injury in a murine model of endotoxemia, aligning with its known cardiovascular benefits. To move beyond phenomenological observations, a comprehensive transcriptomic analysis was pivotal, revealing that Empa’s molecular signature extends beyond its established anti-inflammatory actions. While pathways such as NF-κB signaling were suppressed, the analysis critically unveiled a dominant enrichment of pathways related to cytoskeletal integrity, particularly the “Cytoskeleton in muscle cells” KEGG pathway. This insight directed our investigation towards the fundamental structural and mechanical scaffold of cardiomyocytes, identifying the pathological dysregulation of the Integrin α5 (ITGA5) and Desmocollin-2 (DSC2) adhesion axis as a core feature of septic cardiac injury. We observed that the inflammatory challenge pathologically inverts the homeostatic relationship between these proteins, causing a deleterious upregulation of ITGA5 concurrent with a loss of DSC2, thereby destabilizing both cell–matrix and cell–cell junctions.

To validate these transcriptomic insights and determine the locus of Empa’s action, we conducted a multi-tiered protein-level investigation. Immunohistochemical and Western blot analyses of cardiac tissue confirmed this reciprocal dysregulation in vivo, demonstrating that LPS induces a significant increase in ITGA5 and a concomitant decrease and delocalization of DSC2 from the intercalated disks. Crucially, Empa treatment prevented this pathological remodeling, preserving the expression and proper localization of both adhesion proteins. To dissect whether this protective effect was a direct cardiomyocyte-intrinsic action or a secondary consequence of systemic modulation, we utilized both primary neonatal mouse cardiomyocytes and a human cardiomyocyte cell line. In these isolated systems, devoid of systemic influences, LPS stimulation was sufficient to recapitulate the pathological protein signature. Remarkably, concurrent treatment with Empa directly and effectively abrogated this response, normalizing ITGA5 and DSC2 expression. This conclusive, multi-level evidence unequivocally demonstrates that Empa acts directly on cardiomyocytes to preserve the structural integrity of the ITGA5-DSC2 adhesion axis.

The elucidation of this mechanism carries significant implications, reframing our understanding of Empa’s cardioprotective properties by revealing a fundamental structural preservation effect distinct from previously proposed actions like hemodynamic or metabolic modulation. This finding underscores the critical importance of maintaining cardiomyocyte homeostasis during severe inflammatory stress. Building on this insight, we sought to translate our findings into a potential therapeutic strategy by computationally designing novel protein binders. The binder specifically target ITGA5 was guided by key considerations of therapeutic accessibility and its pathological response. As a transmembrane protein with a large, exposed extracellular domain, ITGA5 is a more accessible target for exogenous binders than DSC2, which is located deep within the desmosomal junction. Moreover, LPS induces a pathological upregulation of ITGA5, making its over-activity a clear target for inhibition, whereas DSC2 is downregulated and mislocalized—a state less amenable to correction with binder agent. Therefore, we want to design a binder that normalizing aberrant ITGA5-mediated signaling could restore the homeostatic balance of the entire adhesion axis. Our computational pipeline successfully yielded binders predicted to engage the intended hotspot on ITGA5. These de novo proteins represent a promising, mechanism-based modality that, either alone or in synergy with Empa, offers a new paradigm for preventing cardiac structural failure in sepsis.

While this study provides evidence for a novel mechanism of Empa, several limitations must be acknowledged that also define critical avenues for future research. A primary limitation stems from its prophylactic design, wherein empagliflozin was administered prior to the septic insult. Consequently, our findings primarily highlight a preventive effect, necessitating future investigations into empagliflozin’s therapeutic efficacy when administered post-sepsis onset, which would more accurately reflect clinical scenarios. Moreover, our use of a single-dose, acute endotoxemia model (via LPS injection) in standard, wild-type male C57BL/6 mice, without unique mutations, was deliberate, aimed at recapitulating the hyperinflammatory early phase of sepsis and its associated acute cardiac dysfunction. While this controlled approach facilitates the dissection of rapid, preventive mechanisms by minimizing confounding variables, we explicitly acknowledge its inability to fully replicate the protracted, polymicrobial nature of human sepsis. Compounding these limitations, cardiac functional assessment was restricted to a single endpoint at 24 h post-LPS, lacking baseline or serial measurements, which consequently precludes analysis of the temporal evolution of hemodynamic parameters. Furthermore, while we have identified the stabilization of the ITGA5-DSC2 axis as a key downstream effect, the precise upstream signaling cascade through which Empa mediates this structural preservation remains to be elucidated. A systematic dissection is also required to delineate the relative contribution of this newly identified structural preservation pathway versus the established anti-inflammatory effects to the overall cardioprotective benefit observed. To bridge these translational and mechanistic gaps, subsequent studies will focus on validating these findings in more complex, clinically relevant sepsis models (cecal ligation and puncture [CLP]), evaluating empagliflozin’s efficacy with therapeutic dosing regimens and serial echocardiographic assessments, and prioritizing the experimental validation of our computationally designed ITGA5 binders. Concurrently, mechanistic studies are essential to identify the upstream regulators of the ITGA5-DSC2 axis, and translational validation in clinical studies examining adhesion molecule expression in septic patients will be critical. Finally, exploring whether this protective mechanism extends to other etiologies of acute cardiac injury would significantly broaden the therapeutic implications of our discovery.

## 5. Conclusions

In conclusion, we identify the stabilization of the ITGA5-DSC2 cardiomyocyte adhesion axis as a novel mechanism underlying the profound cardioprotective effects of empagliflozin in sepsis-induced cardiomyopathy. This discovery not only provides a deeper understanding of Empa’s therapeutic efficacy by highlighting the critical role of structural homeostasis but also paves the way for novel mechanism-based strategies for treating this life-threatening condition.

## Data Availability

The dataset (GSE200598) analyzed during the current study is publicly available in the GEO repository. The source code used for the transcriptomic analysis will be made publicly available on GitHub after the publication of this paper or can be obtained from the corresponding author upon reasonable request. All other data generated or analyzed during this study are included in this published article and its Supplementary Information Files.

## Supplementary Materials

The following supporting information can be downloaded at: https://www.mdpi.com/article/10.3390/cells14181452/s1, Table S1: Echocardiographic Parameters Statistical Comparison; Table S2: Design Metrics and Sequences of Computationally Designed ITGA5 Binders.

## Abbreviations

**Abbreviation**	**Full Name**
α-SMA	α-smooth muscle actin
ANOVA	Analysis of Variance
BCA	Bicinchoninic acid
CRP	C-reactive protein
cTnT	Cardiac troponin T
DAVID	Database for Annotation, Visualization and Integrated Discovery
DSC2	Desmocollin-2
ECM	Extracellular Matrix
ECL	Enhanced chemiluminescence
EF	Ejection Fraction
ELISA	Enzyme-linked immunosorbent assay
Empa	Empagliflozin
FS	Fractional Shortening
GEO	Gene Expression Omnibus
GO	Gene Ontology
HE	Hematoxylin and Eosin
IHC	Immunohistochemistry
i.g.	Intragastrically
i.p.	Intraperitoneal
ITGA5	Integrin α5
KEGG	Kyoto Encyclopedia of Genes and Genomes
LPS	Lipopolysaccharide
NMCMs	Neonatal mouse cardiomyocytes
PCA	Principal Component Analysis
PVDF	Polyvinylidene difluoride
RIPA	Radioimmunoprecipitation assay
SDS-PAGE	Sodium dodecyl sulfate–polyacrylamide gel electrophoresis
SEM	Standard Error of the Mean
SGLT2	Sodium-glucose cotransporter 2
TBST	Tris-buffered saline with Tween 20
