# Supplementary material for: Empagliflozin Preserves Cardiomyocyte Structural Homeostasis via the Stabilization of the Integrin α5–Desmocollin-2 Adhesion Axis in Sepsis-Induced Cardiomyopathy
Source: Cells. 2025 Sep 16;14(18):1452. doi: 10.3390/cells14181452 (PMC12468141; doi:10.3390/cells14181452)
Supplement: Supplementary file 1 [file cells-14-01452-s001.zip › cells-3803008-supplementary.pdf]

**Table S1.** Echocardiographic Parameters Statistical Comparison

| Parameter | Control (n=8) | LPS (n=10) | LPS+EMPA (n=11) | P-value |
|---|---|---|---|---|
| E/A Ratio | 1.554 ± 0.185 | 1.102 ± 0.077 | 1.319 ± 0.083 | <0.001 |
| LVAW;d | 0.784 ± 0.109 | 1.111 ± 0.184 | 0.893 ± 0.142 | <0.001 |
| LVAW;s | 1.144 ± 0.105 | 1.168 ± 0.201 | 1.086 ± 0.160 | 0.502 |
| LVID;d | 3.970 ± 0.317 | 3.435 ± 0.431 | 3.426 ± 0.595 | 0.038 |
| LVID;s | 2.735 ± 0.362 | 3.138 ± 0.428 | 2.706 ± 0.558 | 0.09 |
| LVPW;d | 0.866 ± 0.145 | 0.916 ± 0.204 | 1.045 ± 0.188 | 0.103 |
| LVPW;s | 1.274 ± 0.147 | 1.038 ± 0.258 | 1.276 ± 0.258 | 0.049 |
| EF(%) | 58.971 ± 9.968 | 19.886 ± 4.683 | 41.677 ± 9.759 | <0.001 |
| FS(%) | 31.200 ± 6.942 | 8.762 ± 2.147 | 20.259 ± 5.611 | <0.001 |

Data presented as mean ± standard deviation; Statistical analysis: One-way ANOVA; * p<0.05, ** p<0.01, *** p<0.001;

**Table S2.** Design Metrics and Sequences of Computationally Designed ITGA5 Binders.

| Rank | Design | Protocol | Length | Seed | Helicity | Sequence of ITGA5 binder | MPNN_score | MPNN_seq_recovery |
|---|---|---|---|---|---|---|---|---|
| 1 | ITGA5_CYTO_binder_l75_s658345_mpnn3 | 4stage | 75 | 658345 | -0.3 | APQPSIEELMERYPVFMLTFIWSADDTVTIIWKLISGSEEWREGFYRGLSAEDQAVYDATGVVQQTNTYSPEERG | 1.07 | 0.35 |
| 2 | ITGA5_CYTO_binder_l75_s761192_mpnn1 | 4stage | 75 | 761192 | -0.3 | MLASKEEIDEIFKKARELVEEGAEKMVAYIEEKLKELKDSHISWSWFWLDMDGEKYLVVMLWWGGTIRVSIFQVS | 1.07 | 0.13 |
| 3 | ITGA5_CYTO_binder_l75_s309306_mpnn1 | 4stage | 75 | 309306 | -0.3 | MSKFSPEELKKRTEELEEEYYRVHDEYRRLREEGNLEGYEDLWHEAMRLQLRYMVEKAIYEQALGLEPSTHRMWF | 1.04 | 0.33 |
| 4 | ITGA5_CYTO_binder_l75_s839643_mpnn14 | 4stage | 75 | 839643 | -0.3 | MKVLERQQVWHIVIITRGQSGEMKLLWFWTDVEVDTTLSPEERTKQIDEESTKKSEEELKKLGIDPSTVKSMDHP | 1.21 | 0.3 |
| 5 | ITGA5_CYTO_binder_l75_s839643_mpnn8 | 4stage | 75 | 839643 | -0.3 | MKVLVRQQVWHIVIITRGQSGEMTLLWDWSDVDVDTSLSPEEQTKEIDEKSTAKSREWLASKGIDPSTVKSMDHP | 1.16 | 0.24 |
| 6 | ITGA5_CYTO_binder_l75_s761192_mpnn15 | 4stage | 75 | 761192 | -0.3 | MLPSKEEIDSWFEKVRELMEQGPELALKYLEEKLKSQKDAHISWSWFWLDMDNERYLVVMLWWGGTIRVSIFQVS | 1.27 | 0.22 |
| 7 | ITGA5_CYTO_binder_l75_s761192_mpnn9 | 4stage | 75 | 761192 | -0.3 | MLPSKEEIDEMFEKARELVEEGAEKAKEYLDEWISSQKDAHISWSWFWLDMDGEKYLVVMLWWGGTIRVSIFQVS | 1.17 | 0.22 |
| 8 | ITGA5_CYTO_binder_l75_s839643_mpnn4 | 4stage | 75 | 839643 | -0.3 | MKVLERQQVWHIVIITRGQSGEMKLEWWWSEVEVDSTLSPEEQTKEIDEKSTKLSNEWLKSKGIDPSTVKSMDHP | 1.14 | 0.3 |
| 9 | ITGA5_CYTO_binder_l75_s839643_mpnn16 | 4stage | 75 | 839643 | -0.3 | PKVLVRQQVWHIVIITRGQSGEMRLLWDWFDVDVDSSLSPEEQTQQIDSLSTVKSREWLKSLNIDPSTVKSMDHP | 1.22 | 0.24 |

| | | | | | | | | |
|---|---|---|---|---|---|---|---|---|
| 10 | ITGA5_CYTO_binder_l75_s761192_mpnn12 | 4stage | 75 | 761192 | -0.3 | MLMSKQELDDMFEEFRRLVSEGAEKAIAYLEQWAKSHSDSHLSWSWFWLDMDGERYLVVMVWWGGTIRVSIFQVS | 1.23 | 0.18 |
| 11 | ITGA5_CYTO_binder_l75_s761192_mpnn8 | 4stage | 75 | 761192 | -0.3 | MLISEEELKKLFEEARKLVEEGAEKALEYLEKEIKKHKDSHLSWSWFWLDMDGEKYLVVMVWWGGTIRVSIFQVS | 1.16 | 0.16 |
| 12 | ITGA5_CYTO_binder_l75_s213015_mpnn1 | 4stage | 75 | 213015 | -0.3 | SMKVEMTVRVVWTWNGVRMELTVKQGDRTVTSTKHVYYENGKWWQKVTTRVEEKGKVTEKVEEYEWDGTKWVPVK | 1.11 | 0.41 |
| 13 | ITGA5_CYTO_binder_l75_s839643_mpnn2 | 4stage | 75 | 839643 | -0.3 | SKKLVRQQVWHIVIITRGQSGEMELLWDWTVVEVDTTLSPEEQTKQIDEKSTAQSKAWLATLGIDPATVKSMDHP | 1.1 | 0.35 |

4stage: design with logits->softmax(logits)->one-hot->pssm_semigreedy (default, extensive); MPNN_seq_recovery: MPNN sequence recovery of original trajectory;  MPNN_score: MPNN sequence score;
